# Functionalised zinc oxide nanowire gas sensors: Enhanced NO_2_ gas sensor response by chemical modification of nanowire surfaces

**DOI:** 10.3762/bjnano.3.43

**Published:** 2012-05-02

**Authors:** Eric R Waclawik, Jin Chang, Andrea Ponzoni, Isabella Concina, Dario Zappa, Elisabetta Comini, Nunzio Motta, Guido Faglia, Giorgio Sberveglieri

**Affiliations:** 1 School of Chemistry, Physics & Mechanical Engineering, Queensland University of Technology, 2 George Street, 4000 Brisbane, Australia; 2 SENSOR Lab, CNR-IDASC & Brescia University, Chemistry & Physics Department, Via Valotti 9, 25133 Brescia, Italy

**Keywords:** gas sensor, nanowire, tris(hydroxymethyl)aminomethane, self-assembled monolayer, zinc oxide

## Abstract

Surface coating with an organic self-assembled monolayer (SAM) can enhance surface reactions or the absorption of specific gases and hence improve the response of a metal oxide (MOx) sensor toward particular target gases in the environment. In this study the effect of an adsorbed organic layer on the dynamic response of zinc oxide nanowire gas sensors was investigated. The effect of ZnO surface functionalisation by two different organic molecules, tris(hydroxymethyl)aminomethane (THMA) and dodecanethiol (DT), was studied. The response towards ammonia, nitrous oxide and nitrogen dioxide was investigated for three sensor configurations, namely pure ZnO nanowires, organic-coated ZnO nanowires and ZnO nanowires covered with a sparse layer of organic-coated ZnO nanoparticles. Exposure of the nanowire sensors to the oxidising gas NO_2_ produced a significant and reproducible response. ZnO and THMA-coated ZnO nanowire sensors both readily detected NO_2_ down to a concentration in the very low ppm range. Notably, the THMA-coated nanowires consistently displayed a small, enhanced response to NO_2_ compared to uncoated ZnO nanowire sensors. At the lower concentration levels tested, ZnO nanowire sensors that were coated with THMA-capped ZnO nanoparticles were found to exhibit the greatest enhanced response. Δ*R*/*R* was two times greater than that for the as-prepared ZnO nanowire sensors. It is proposed that the Δ*R*/*R* enhancement in this case originates from the changes induced in the depletion-layer width of the ZnO nanoparticles that bridge ZnO nanowires resulting from THMA ligand binding to the surface of the particle coating. The heightened response and selectivity to the NO_2_ target are positive results arising from the coating of these ZnO nanowire sensors with organic-SAM-functionalised ZnO nanoparticles.

## Introduction

Semiconductor gas sensors have been extensively investigated for practical applications such as the detection of gas leaks and the environmental monitoring of gaseous pollutants. Since the earliest reports in this field, research efforts were focussed on improving gas response, selectivity, and sensor stability, and on their practical use, yet further innovations in the semiconductor gas-sensing field are still in demand [1–3]. Impedance-semiconductor gas sensors typically operate at temperatures greater than 200 °C [4–5]. High operating temperatures are generally required to maximise the sensor response to target gases, either to activate the semiconductor surface towards chemisorption or else to ensure heterogeneous catalysis of a high proportion of target gas molecules at the sensor surface. High-temperature operation also ensures the complete desorption of gaseous species following transduction. Maintaining a semiconductor gas sensor at a stable temperature higher than 200 °C requires a stable and consistent power source, drawing on a high operating voltage and current. For certain applications maintaining the high operating temperature can have drawbacks, especially when high sensor power consumption is undesirable, such as when photovoltaics are the desired power source. A case in point is the monitoring of gas emissions remotely, in outdoor environments where mains power may be unavailable. For this application, highly responsive, low-power and thus low-temperature gas sensors would be advantageous.

Both the structural and physicochemical properties of metal-oxide films utilised in solid-state chemical sensors have proven to strongly affect the gas response in these devices. Not only do simple structural elements such as grain size play a significant role in gas response, but also crystallite shape, crystallographic orientation, film agglomeration, phase composition and surface architecture [5]. In terms of the targeted optimisation of gas-sensor characteristics, surface engineering is potentially a powerful instrument for the control of gas response. To date, designers have mainly focused on doping the metal oxide by means of metal catalyst nanoparticle additives, and decreasing the crystallite (grain) sizes or intercrystallite neck dimensions to the nanometre scale. Chemical functionalisation as an approach to modify the response of semiconductor surfaces towards different gases has not been examined to anywhere near the same level of detail. Coating of the semiconductor with a sensitising molecule layer could enhance surface reactions or modify the surface chemistry and, hence, improve sensor sensitivity and specificity to a particular gas. Although such chemical functionalisation of impedance-based gas sensor surfaces is often avoided due to the possibility of poisoning effects which may occur, akin to poisoning of heterogeneous catalyst surfaces, functionalisation can sometimes have a positive effect [6]. Self-assembled monolayers (SAMs) have been shown to effectively modify the surface physics and chemical properties of metals and metal-oxide materials [6]. Previously, when foreign receptors were introduced into MOx sensor grains, sensitizing actions were observed, particularly when they modified the work function and surface space-charge layer of the sensor material [7]. Another significant effect that may arise through organic SAM functionalisation of the semiconductor surface is a chemical effect. Since only the outermost 5 Å of a surface completely determines its chemical properties, that is whether it is hydrophobic, hydrophilic, acidic, or basic for example, surface functionalisation with an organic molecule may be expected to change the relative rates of diffusion of gaseous species to the surface of the semiconductor and alter the reaction processes that occur. An organic SAM can act as a functional group in nanowire chemical and biological sensors [6,8]. In terms of sensor response, although a decreased number of “active” sites for chemisorption may arise through chemical functionalisation by an organic layer, the effect may be offset by increased rates of gas decomposition or reduced interference caused by moisture or other species present in a gas stream. It should be noted that since chemisorption involves electronic charge transfer, functionalisation of the surface of a metal-oxide semiconductor gas sensor with an organic monolayer will strongly influence the electronic properties of the surface. Transfer of electron density into the semiconductor will reduce the depletion layer, which is an effect that is likely to modify the chemiresistor response significantly [3].

ZnO is one of the most widely studied materials, due to its promising optical, optoelectronic and piezoelectric properties [9–10]. Furthermore, ZnO materials can be reliably synthesised in a variety of different nanostructured forms, such as nanowires, nanoribbons, nanobelts and as tetrapods [11], and their potential use in NO_2_ gas sensing in these forms is well known [12–13]. In this study we investigated the effects that two very different types of organic ligands imposed on the sensitivity and response of semiconductor gas sensors based on zinc oxide nanowires. Significantly, we chose to examine the response of *the same* ZnO nanowire sensors to target gases before and after functionalisation rather than to compare different sensors prepared in the same batch, in order to eliminate the possibility of differences in response caused by sensor batch variations being misconstrued as a result of the functionalisation process. The first ligand studied was dodecanethiol (DT), which can readily form a self-assembled monolayer at the ZnO surface simply by exposure of the nanowires to an ethanolic solution containing the thiol. The long DT hydrocarbon chains were expected to create a strongly hydrophobic surface. Similarly ZnO nanowire sensors were functionalised by tris(hydroxymethyl)aminomethane (THMA), and the response of THMA-functionalised sensors was compared to that of the unfunctionalised ZnO nanowire devices. Since the working principle of an oxide-semiconductor gas sensor involves the receptor function that is played by the surface of each oxide grain and the transducer function that is played by each grain boundary [3], the self-assembly of DT and THMA monolayers on the surface of the oxide was expected to modify both functions. The performances of both ZnO nanoparticle (ZnO NP) sensors and also ZnO nanowire (ZnO NW) sensors coated with a low density of DT-, or THMA-functionalised ZnO nanoparticles were examined and compared to nanowire-only devices to test the influence of grain-boundary effects on the gas response. We investigated the chemiresistor response towards ammonia, nitrous oxide and nitrogen dioxide.

## Results and Discussion

**Figure 1:**
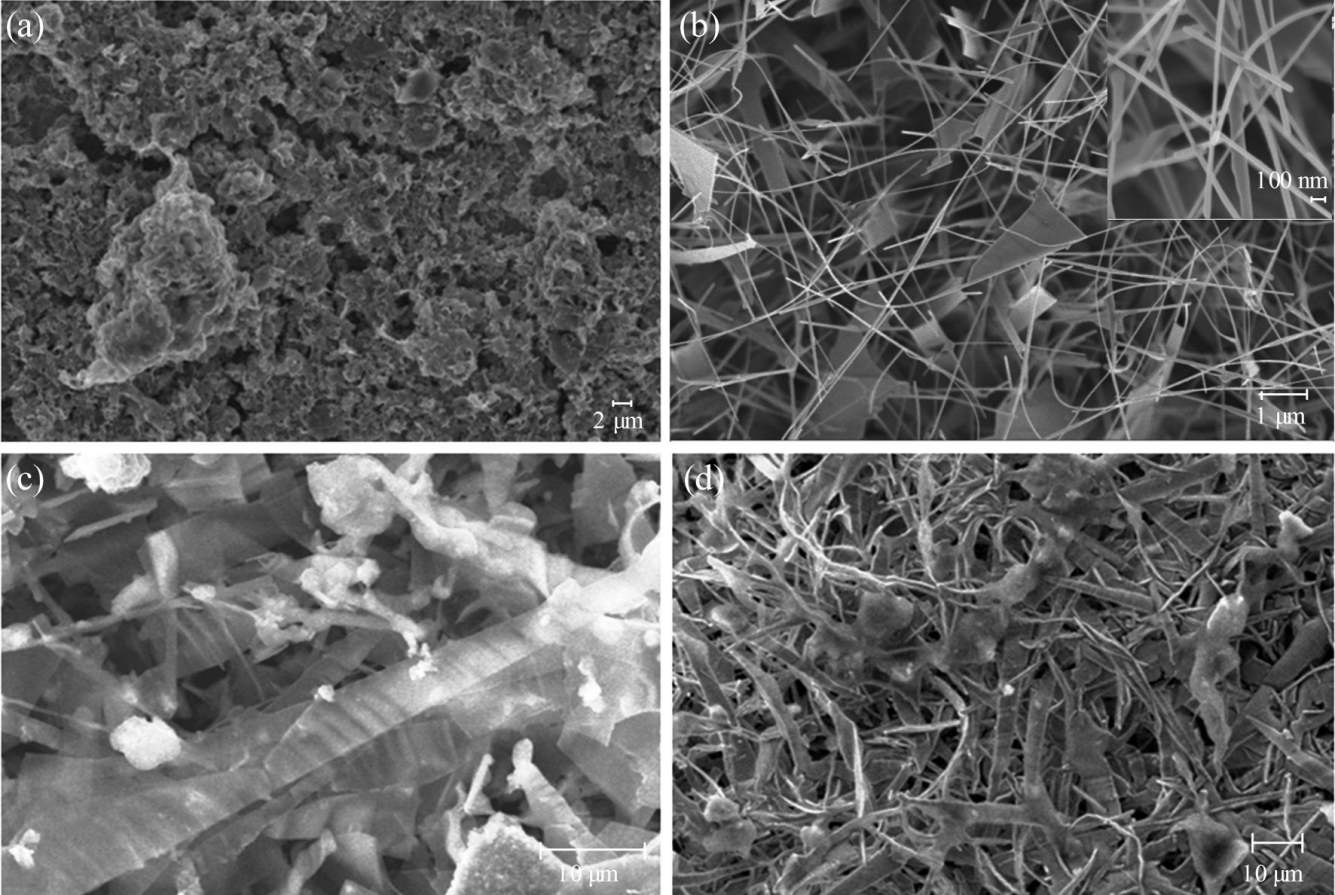
Scanning electron microscopy images of (a) the drop-cast ZnO nanoparticle sensor surface; (b) the pure ZnO nanowire sensor surface (inset: higher magnification FESEM image of ZnO NWs); (c) DT-ZnO NP + ZnO nanowire sensor surface; and (d) THMA-ZnO NP + ZnO nanowire sensor surface (note the change in scale).

**Figure 2:**
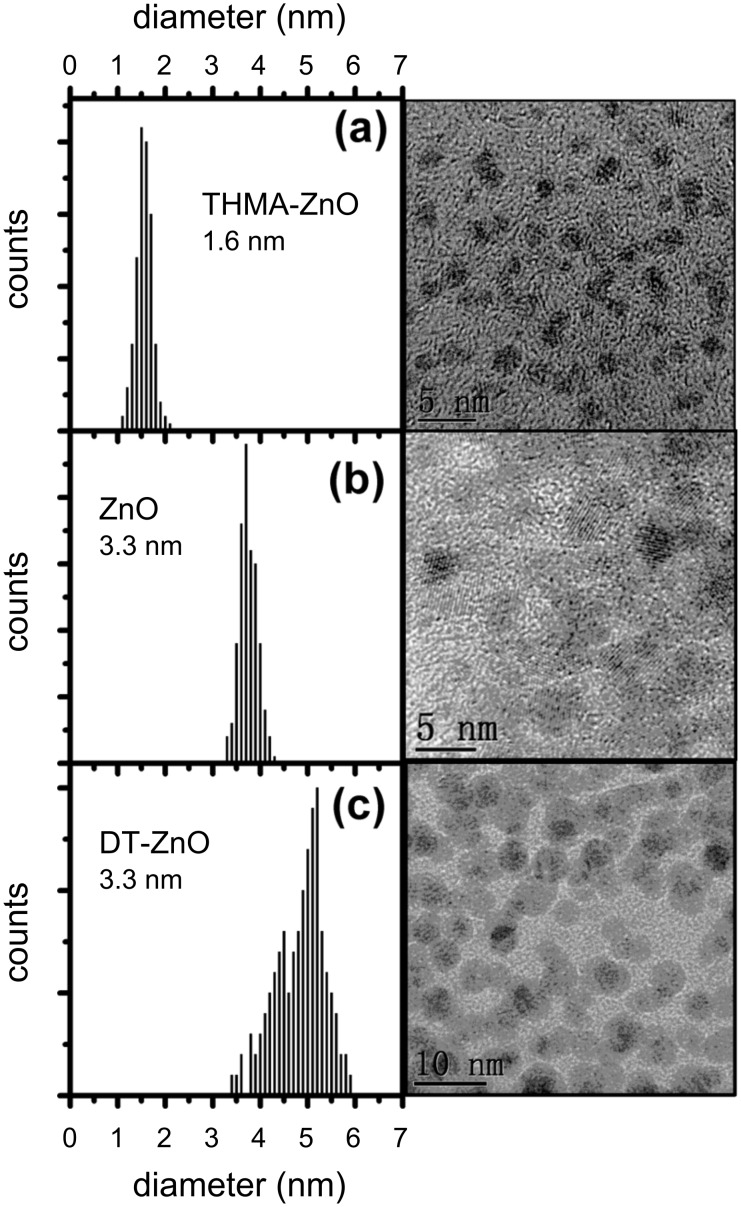
Transmission electron microscopy images and size-distribution analyses of ZnO nanocrystals after heating under reflux in ethanol for 30 min, for (a) THMA-functionalised ZnO nanoparticles; (b) ZnO NPs; and (c) DT-functionalised ZnO NPs.

The morphology, surface roughness and evenness-of-coating of the ZnO nanowire sensors were examined by scanning electron microscopy. SEM images of each sensor form are given in Figure 1. The SEM image in Figure 1a is of a drop-cast nanoparticle-based sensor. The nanoparticle films formed as high-surface-area, porous coatings, which were evenly deposited over the sensor support, including the metal contacts. When these sensors were tested for gas response, however, the conductivity of the ZnO NP films, as produced, proved too low to be useful in the gas-sensor configuration tested. The ZnO matrix deposition process clearly led to agglomeration of the primary ZnO particles into aggregates, some with micrometre dimensions. Such agglomeration of small crystallites into larger masses tends to reduce the gas permeability through the matrix [14]. It also increases the influence of the interagglomerate contact resistance on the gas response of the sensors. Analysis of transmission electron microscope images of these ZnO materials reveals that the primary particle size of these nanoparticle-based sensors was in the range 1 to 6 nm (Figure 2), yet the background resistance of these sensor elements was very high, presumably due to inter-agglomerate contact effects [14]. Strong agglomeration thus appeared to offset any of the advantages in terms of sensitivity that could be obtained through the use of small ZnO grains (crystallites) with these sensors. In the case of ZnO nanoparticle sensors in the size regime used here, it has been demonstrated previously that a significant response to low (2–10 ppm) NO_2_ concentrations requires an operating temperature of 290 °C to elicit the maximum response of the material [15]. Since we were examining the potential useful effects that a ligand shell surrounding the nanoparticles could have upon the response to NO_2_ at 190 °C, it was unsurprising that only a very low transient response was observed with the nanoparticle sensors.

In contrast to the case of drop-cast ZnO nanoparticle sensors, the nanowire samples (Figure 1b) were evenly deposited as an open structure, containing in this case a mixture of thin sheets and nanowire filaments. The individual nanowires typically had dimensions of 5–10 μm in length and approximately 50 nm in diameter (see inset to Figure 1b). The low resistivity of these devices, as made, arises from the high degree of crystallinity and the high aspect ratio of the nanowires. This particular structure thus offers preferential, low-resistance paths to charge carriers, with percolative conduction paths featuring a much-reduced number of crystallite interfaces with respect to their nanoparticle counterparts. Organic functionalisation of nanowire sensors identical to that shown in Figure 1b had no visible effect on the nanowire structures as seen by FESEM (either with DT or THMA). Finally, the SEM images of nanoparticle-coated samples in Figure 1c and Figure 1d show that the pure ZnO-based samples were effectively coated, yielding thicker, functionalised ZnO NP filaments and platelets. In each case ZnO NP agglomerates were also attached to the nanowire samples, sometimes contacting several plates or wires.

**Figure 3:**
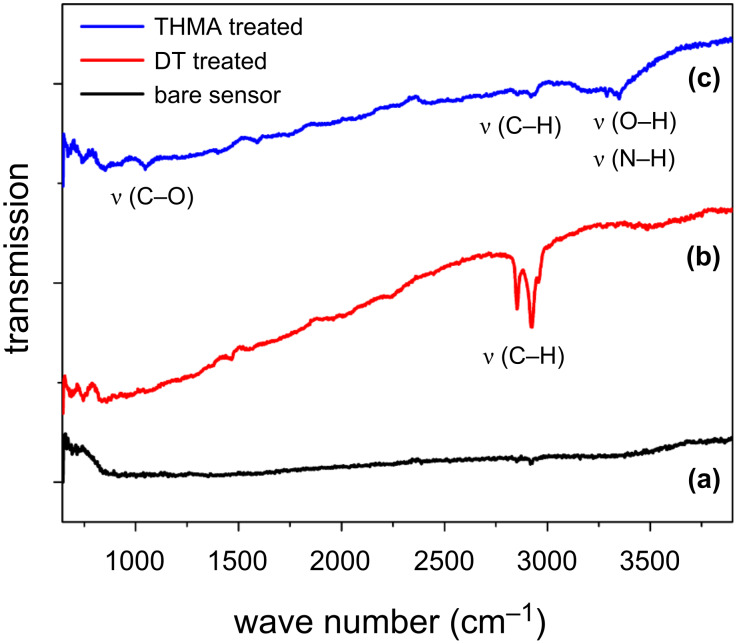
FTIR spectra of (a) pure ZnO nanowire sensor; (b) dodecanethiol-coated ZnO nanowire sensor; and (c) THMA-coated ZnO nanowire sensor.

Ex situ functionalisation of NP- and NW-powder ZnO samples by either DT or THMA was confirmed by FTIR measurements. The FTIR spectra given in Figure 3 are of a ZnO nanowire sensor sample and samples that had been exposed to either 10 mM dodecanethiol or THMA solution for 24 h, followed by repeated washing with ethanol in order to remove possible excess surface-adsorbed organics. These samples were then dried in air. Evidence for successful the functionalisation of ZnO by DT can be seen by comparing the FTIR spectra in Figure 3. In Figure 3b, FTIR absorption peaks originating from the dodecanethiol C–H symmetric and asymmetric stretch vibrational modes can be clearly seen at 2850 and 2920 cm^−1^, respectively, which indicates that functionalisation of ZnO by DT in this way was successful. The dodecanethiol peaks were completely absent in the pure ZnO FTIR spectrum of Figure 3. The presence of a C–O stretching peak near 1100 cm^−1^ and an N–H peak near 3300 cm^−1^ in the spectrum of a THMA-functionalised ZnO nanowire sensor Figure 3 also revealed that THMA adsorbed to the ZnO nanowires.

**Figure 4:**
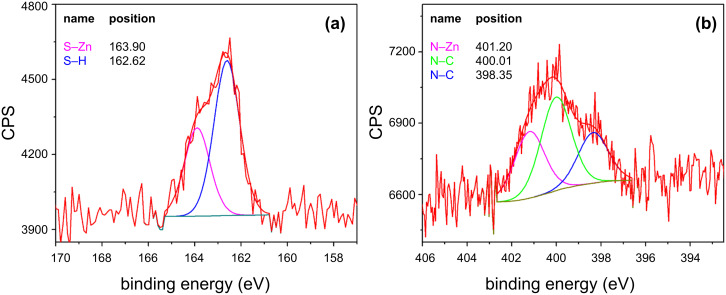
XPS spectra of (a) the sulfur peak of DT-functionalised ZnO NW sensor surface and (b) the amide peak of THMA-functionalised ZnO NW sensor surface.

**Figure 5:**
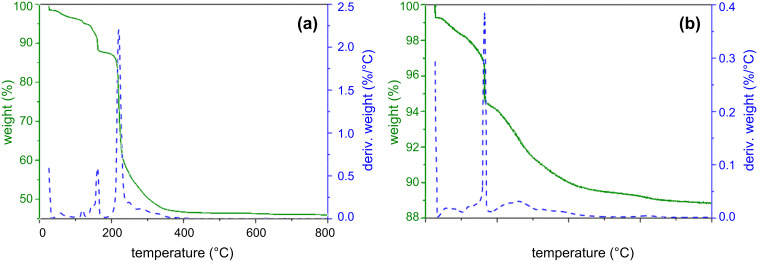
TG and DTG of (a) DT-coated and (b) THMA-coated ZnO obtained in air at 5 °C·min^−1^.

The FTIR evidence for the successful functionalisation of ZnO sample surfaces by DT and THMA was confirmed by both XPS and thermogravimetry. The XPS survey of the DT-functionalised ZnO NW sensor surfaces given in Figure 4a provides further evidence for the successful chemisorption or ligand attachment of dodecanethiol to the ZnO NW surfaces. A peak corresponding to the Zn–S bond between the dodecanethiol ligand and the ZnO surface could readily be distinguished at 164.4 eV with these samples, while sulfur peaks in the XPS spectrum of the unfunctionalised sensors were completely absent. Similarly THMA-functionalisation led to a multicomponent nitrogen peak, which could be fitted to the C–N bond in THMA occurring at 400.2 eV and also a N–Zn bond between THMA and ZnO at 401.7 eV, as shown in Figure 4b. Similar amine-based peaks were also absent from the XPS spectra originating from pure ZnO NW sensor surfaces. The thermogravimetric results given in Figure 5 were also used to determine the temperature range that the dodecanethiol-coated or THMA-coated sensors could operate in without there being a significant breakdown of the organic monolayer coating. Inspection of Figure 5 reveals that only minor mass-loss peaks occurred between room temperature and 200 °C, corresponding to desorption of surface-adsorbed water and species such as surface acetate ligand residues, which remained after the zinc oxide synthesis and washing procedures [16]. The major mass-loss peak, accounting for 40% of the original mass, presumably corresponding to desorption and break down of the dodecanethiol monolayer, occurred at approximately 225 °C. This desorption temperature for dodecanethiol is significantly lower than that reported by Sadik et al. who used XPS to investigate the functionalization of O-terminated (desorption occurred at 350 °C) and Zn-terminated (400 °C desorption) zinc oxide surfaces [17]. The difference in these values can be ascribed to the fact that our materials were prepared in nanocrystalline rather than single-crystal form and therefore a range of different ZnO crystal surfaces were exposed rather than a single surface, but more importantly, the fact that the XPS study was performed in ultrahigh vacuum rather than in dry air. Taking the TG results into account, a sensor operating temperature of 190 °C was chosen for all gas-response tests.

**Figure 6:**
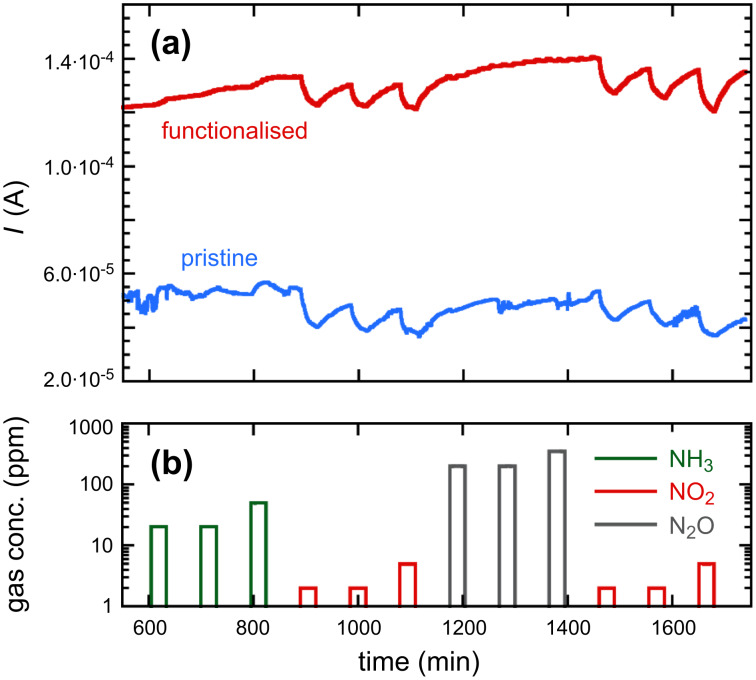
Dynamic response of the same ZnO nanowire sensor, (a) before and after THMA functionalization and (b) during exposure to different concentrations of NH_3_, N_2_O and NO_2_.

Gas sensing measurements for the various ZnO samples with different morphologies and compositions were performed for the gases ammonia, nitrous oxide and nitrogen oxide. The sensors were maintained at the operating temperature of 190 °C in dry air until a stable sensor resistivity was reached, then the sensors were each exposed, in turn, to ammonia, nitrogen dioxide and nitrous oxide. The gas response results for the pure ZnO nanowire samples, and also after the same sensor surface had been functionalised by THMA, are both given in Figure 6. The results for the pristine and THMA-functionalised ZnO nanowire sensors exhibited similar behaviour upon exposure to the different gases. In both cases there was almost no response upon exposure to the reducing gas NH_3_. Likewise these sensors did not appear to show a significant response when exposed to nitrous oxide, even for high concentrations of 300 ppm of N_2_O in the gas stream. In contrast to these results for NH_3_ and N_2_O, exposure of the nanowire sensors to the oxidising gas NO_2_ produced a significant and reproducible response. This result is consistent with the results obtained with other metal-oxide gas sensors: NO_2_ is in general much easier to detect than NH_3_ and N_2_O; NO_2_ is frequently sensed at a ppb level, NH_3_ at a ppm level, see for example Ponzoni et al. [18], while N_2_O is usually sensed at a hundreds of ppm level [19]. So, our results with pure ZnO here clearly reflect the different reactivity of these gaseous molecules with metal oxides. Furthermore, the response of pure ZnO to NH_3_ is usually only enhanced at high temperature (around 300 °C) [20], but this is not compatible with the organic coating, which would be damaged/desorbed at such a high temperature. ZnO and THMA-coated ZnO nanowire sensors both readily detected NO_2_ down to concentrations of 2 ppm. To confirm that the response of the nanowire samples to NO_2_ was reproducible and that no poisoning effects occurred at the sensor surfaces, after exposure to N_2_O, the sensors were re-exposed to NO_2_. It can be seen by inspection of Figure 6 (a), that the response to NO_2_ was unchanged.

The gas response of the *same* ZnO sensors pre- and post-functionalisation with THMA was significant and could be readily measured, although there appeared to be no clear indication of a significant change in NO_2_ sensitivity after THMA functionalization. Furthermore, we note that the baseline conductivity of these sensors was not significantly changed. In contrast, in the case of the DT-functionalised sensors, surface reaction with the thiol (confirmed by FTIR) raised the conductivity of each individual sensor above the measurement range of our instrument (which corresponds to a minimum conductance value of *G*_max_ = 10 mS). We conclude from this that chemisorption of the thiol significantly increased the density of electrons present in the ZnO NW conduction band. We note that ZnO NW sensors modified by DT-functionalised or THMA-functionalised ZnO NPs showed a small increase in baseline conductivity, as can be seen in Table 1.

**Table 1:** Average response of all ZnO NW samples upon exposure to 2 ppm NO_2_.

**code**	**before functionalisation**	***G*_0_ (S)**	**after functionalisation**	***G*_0_ (S)**	***G*_0,after_**/***G*_0,before_**

3a	pure	2.53·10^−3^	+ DDT	out of range	(>3.95)
3b	pure	4.66·10^−3^	+ DDT	out of range	(>2.15)
3c	pure	9.87·10^−3^	+ DDT	out of range	(>1.01)
3d	pure	4.48·10^−3^	+ THMA	6.99·10^−3^	1.56
3e	pure	4.52·10^−3^	+ THMA	7.67·10^−3^	1.70
7a	pure	2.57·10^−4^	+ THMA	6.13·10^−4^	2.38
7b	pure	2.06·10^−4^	+ ZnO NP + DDT	1.80·10^−4^	0.87
7c	pure	1.83·10^−4^	+ ZnO NP + DDT	3.15·10^−4^	1.72
8a	pure	9.25·10^−5^	+ ZnO NP + DDT	1.41·10^−4^	1.52
8b	pure	3.40·10^−4^	+ ZnO NP + THMA	4.29·10^−4^	1.26
8c	pure	8.56·10^−5^	+ ZnO NP + THMA	1.30·10^−4^	1.51
8d	pure	4.48·10^−5^	+ ZnO NP + THMA	8.07·10^−5^	1.80

Overall, the pure and THMA-functionalised ZnO NW sensors proved to be effective for the detection of the oxidising gas NO_2_ (Figure 6). It is well known that ZnO adsorbs atmospheric oxygen to form adsorbed O_2_^−^, O^−^ and O^2−^ species and that these electrons are drawn from the ZnO conduction band as a consequence of this adsorption. Adsorbed O_2_^−^ is stable below 100 °C, O^−^ is the adsorbed species in highest concentration between 100 and 300 °C, while O^2−^ is prevalent above 300 °C [21–22]. At the sensor operating temperature of 190 °C, the strongly oxidising gas NO_2_ also depletes ZnO of electrons upon chemisorption, leading to a reduced conductivity after the gas exposure, according to the following process:

[1]
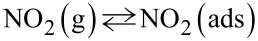


[2]
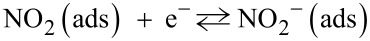


One approach to the use of organic SAMs to enhance gas response is to use them to generate extra oxygen vacancies and defects in the MOx sensor surface. This has been successfully demonstrated previously for the case of a ZnO nanobelt oxygen gas sensor, by heating the sensor to temperatures at which desorption and decomposition of the organic SAM occurred [6]. This is not likely to be the case here, however. The approach in this study was slightly different. The sensors were heated close to the SAM decomposition temperature, which was confirmed using TG (Figure 5), but not so far that significant SAM decomposition was likely to occur. Temperature-programmed desorption (TPD) experiments have shown that chemisorbed thiolates remain stable on the ZnO surface up to approximately 500 K (227 °C) [23]. Similarly for the case of amines like THMA, adsorption leads to a Lewis acid/base interaction [24], and our TG results show that for THMA, no significant decomposition or desorption occurred at 190 °C. Since a thick and impervious monolayer coating would prevent any contact between the gas and the sensor surface, leading to little to no response, the organic layer must in each case be porous enough to allow gas molecules to pass to the semiconductor surface and to interact with surface-adsorbed oxygen according to the mechanisms outlined in Equation 1 and Equation 2. There must also be enough defects or reactive sites at the semiconductor surface initially, for the THMA molecules (and presumably for DT molecules) to react at defects or reactive sites and inject negative charge carriers into the material. Thus functionalisation has the opposite effect to O_2_ (and NO_2_) adsorption. Electrons are injected into the conduction band of the semiconductor by THMA, just as they are for DT, albeit to a lesser extent.

**Figure 7:**
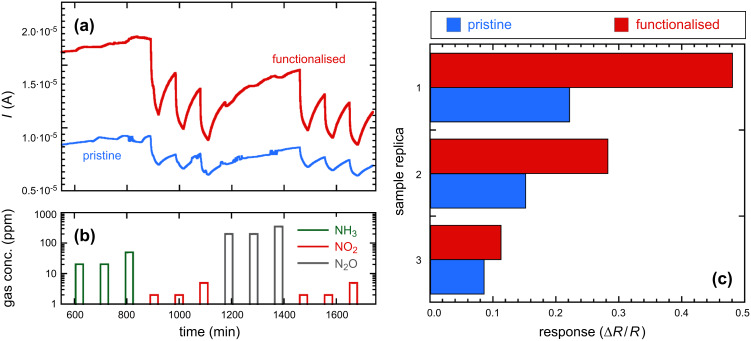
Dynamic response of the same ZnO nanowire sensor, (a) before and after coating with ZnO nanoparticles and THMA; (b) during exposure to different concentrations of NH_3_, N_2_O and NO_2_; and (c) response Δ*R*/*R* exhibited by three sensor replicas before and after THMA functionalization to 2 ppm of NO_2_.

The dynamic responses of the THMA-functionalised ZnO-nanowire-based chemiresistor gas sensors are given in Figure 7a and Figure 7b, as operated at the comparatively low temperature of 190 °C. It is interesting to note that all three of the replicas of these sensors possessed a consistent enhanced sensitivity toward NO_2_ after they had been coated with the nanoparticles and THMA. This is of interest from a practical point of view due to the enormous number of different types of organic molecules that could in principle be used to enhance sensor response. Nanoparticle size and density-of-coverage are other avenues that could be explored in this context. A consistent enhancement of response with these capped nanoparticle-functionalised resistive gas sensors toward particular gases, may allow them to operate at lower temperatures and so reduce the power consumed by these devices. Perhaps just as intriguing as the potential practical applications of this type of coating, is the possible mechanism by which the enhanced gas response was achieved, by attachment of the particles to the surface of the sensor.

Of the two organic monolayers investigated, DT and THMA, THMA was likely to be the stronger capping ligand for ZnO, as demonstrated by the smaller nanoparticle size and nanoparticle distributions produced when THMA was introduced to the zinc acetate synthesis route (Figure 2). THMA is clearly able to displace any surface adsorbed acetate on the ZnO NPs and stifle further particle growth during synthesis. This being the case, THMA is also likely to bind strongly to ZnO nanowire surfaces. The presence of the three hydroxyl groups in THMA ensures multidentate binding to the ZnO surface and is likely to facilitate hydrogen-bonding type interactions with surface-adsorbed hydroxyls under our surface-functionalisation (and gas-testing) conditions. In terms of an electronic effect, THMA (and DT) binding led to an increase in free-electron carrier concentration in ZnO, reflected in the increased conductivity of the functionalised nanowire gas sensors (Table 1). In terms of gas response, there are several ways in which a gas sensor can achieve enhanced response in the case of ZnO nanowire sensors. For instance it is well known that gas sensitivity to NO_2_ is linearly proportional to oxygen-vacancy-related defects [25]. THMA-SAMs appeared to have little effect on Δ*R*/*R* with NO_2_ exposure, so in this case the surface-ligand binding did not appear to affect sensor response by changing this parameter significantly. Since the gas-sensor configuration with the most enhanced gas response was the THMA-ZnO-nanoparticle-coated configuration, it appears that some property arising through the coating with the nanoparticles influenced the gas response to NO_2_ in a positive way, modulating the response by increasing the resistance through the ZnO nanowire contacts.

A reasonable explanation for this can be suggested upon comparison of Table 1 with the results shown in Figure 6 and Figure 7. Due to the reduced size, about 1.3 nm, which is comparable with the depth of the depletion region, the nanoparticles are likely to be fully depleted of electrons, different to the case of nanowires, which feature a depleted surface layer but also possess an unaltered “bulk” core due to their much larger diameter (about 50 nm, from Figure 1 inset). Looking at the overall results obtained with all the sensor replicas, as shown in Table 1, the more effective baseline conductance increase obtained after coating with THMA-ZnO nanoparticles compared to the THMA-coating, can be explained in terms of a preferential charge-carrier injection from THMA into depleted nanoparticles instead of nanowires. On the other hand, exposure to NO_2_ would modify the charge-carrier equilibrium between THMA and the ZnO nanowire, with THMA injecting additional carriers to balance the effects of NO_2_, thus quenching, in part, the overall sensor response to that gas. Following this scheme, it is not surprising that nanowires feature an almost unaltered response to NO_2_ after being coated with THMA. In the case of the THMA-ZnO nanoparticles coating, the presence of such small nanoparticles is expected to mitigate such a quenching effect by preventing a large charge injection into nanowires. The overall result from the THMA- and nanoparticles-functionalised cases is a response increase in these samples, consistent with the results shown in Figure 7.

## Conclusion

In this study the dynamic gas response of resistive gas sensors formed from functionalised ZnO nanowires was examined. The response of the nanowire sensors when functionalised with organic molecule layers of dodecanethiol, tris(hydroxymethyl)aminomethane and also with DT, or THMA-functionalised ZnO nanoparticles was compared for ammonia, nitrous oxide and nitrogen dioxide. Of the three gases examined, only the reducing gas NO_2_ generated a significant response in these sensors. Significantly, rather than averaging the results of several different sensors made in a single batch, the responses of the *same* individual sensors were compared pre- and postfunctionalisation. Any changes in response could thus be attributed to the functionalisation step. Apart from a slight change in baseline conductivity, the gas response Δ*R*/*R* of DT-functionalised NP-modified ZnO NW sensors did not show any significant enhancement (or poisoning effect) when exposed to NO_2_. In contrast to this, ZnO nanowire sensors coated with a thin layer of very small THMA-functionalised nanoparticles (average nanoparticle diameter 1.3 nm) elicited up to 2× enhancement in Δ*R*/*R* toward very low, 2 ppm, concentrations of NO_2_ following the nanoparticle-coating step. The results obtained demonstrate that the modification of metal-oxide surfaces, such as ZnO nanowires, with nanostructured materials containing organic monolayers, can tune the electronic and interfacial properties of these materials, and in the case of gas sensors, has the potential to enhance the gas response significantly.

## Experimental

Zinc oxide nanowire films were grown on alumina substrates for sensing tests (2 mm × 2 mm × 0.25 mm). The ZnO nanowires were grown from the vapour phase by using the evaporation–condensation technique. Pure Zn precursor powder was placed at the centre of an alumina tube and then the tube temperature was raised above the Zn decomposition temperature of 600 °C. A controlled flow of inert argon gas was maintained during the decomposition and the overall pressure was maintained at several hundreds of mbar. The temperature gradient downstream of the gas flow promoted condensation of metal cations on clean alumina substrates, which interacted with residual oxygen to give ZnO nanowires [13]. The stabilised samples were then provided by interdigitated Pt electrodes deposited by RF magnetron sputtering, while on the back side a Pt meander was deposited to act as heater (by Joule effect) and temperature sensor. Ex situ functionalisation of dodecanethiol-coated nanowire samples was accomplished by immersing the completed sensors for 24 h in a 10 mM solution of dodecanethiol in ethanol. Tris(hydroxymethyl)aminomethane-functionalised ZnO nanowires and nanoparticles were similarly functionalised by immersing the completed sensors for 24 h in 10 mM ethanolic solutions. The functionalisation of ZnO by this procedure was confirmed by FTIR and XPS measurements. Prior to functionalisation with ligands or ligand-capped ZnO nanoparticles and the gas-sensing tests, all nanowire samples were stabilised by annealing at 450 °C for 25 d.

Ligand-capped ZnO NPs were all synthesised by the wet-chemical method. All were produced by hydrolysis of anhydrous zinc acetate (Zn(CH_3_CO_2_)_2_ or Zn(Ac)_2_, 99.99%), in the presence of base (sodium hydroxide) in absolute ethanol. Ligands *n*-dodecanethiol (C_12_H_25_SH, or DDT, 99%) and tris(hydroxymethyl)aminomethane (C_4_H_11_NO_3_ or THMA, 99%) were added as required. All chemicals were used as received from Aldrich without further purification or distillation. For example, in a typical synthesis, Zn(Ac)_2_ (0.5 mmol, 92 mg) and THMA (0.2 mmol, 24 mg) were added into absolute ethanol (30 mL) under stirring. Then the mixture was heated at around 80 °C for 1 h to dissolve Zn(Ac)_2_ and THMA. Following complete dissolution of the precursors, a NaOH/ethanol solution (20 mL, 0.05 M) was injected into the hot solution and then the mixture was heated under reflux for a further 72 h. The obtained cloudy solution was centrifuged and rinsed with deionized water and ethanol to remove byproducts. For the synthesis of DT-ligand-capped ZnO samples, THMA was simply replaced by DT. For comparison, bare ZnO NPs were synthesised in the same procedure without the use of capping ligands. The morphology and size of all nanoparticle samples was determined by transmission electron microscope (TEM, JEOL-2100) with an accelerating voltage of 200 kV. For TEM experiments, the specimens were prepared by deposition of a dilute solution of the colloid onto a carbon-coated copper grid and drying at room temperature.

Thermogravimetric (TG) measurements were performed on an SDT 2960 TA® Instruments model at 5 °C·min^−1^ over a temperature range from 24 °C (room temperature) up to 1000 °C under a dry air flow of 100 cm^3^·min^−1^. Derivative thermogravimetric (DTG) curves were generated with approximately 2000 points. Samples were characterised by scanning electron microscopy (SEM) (Zeiss SUPRA 40). FTIR measurements on organic-functionalised ZnO were performed at room temperature by using a Spectrum 1000 FTIR spectrometer. X-ray photoelectron spectroscopy (XPS) measurements of ZnO nanowires were measured before and after functionalisation by either DT or THMA, to confirmed the presence of the ligands on the ZnO nanowire surfaces after the functionalisation, washing and low-temperature anneal had been performed, but prior to gas sensing, by using a Kratos Axis ULTRA XPS incorporating a 165 mm hemispherical electron-energy analyser. Monochromatic Mg Kα X-rays (1253.6 eV) at 150 W (15 kV, 10 mA) were used as the incident radiation. Multiplex high-resolution scans were achieved at an analyser pass energy of 20 eV, in steps of 0.10 eV.

Characterisation of the electrical and gas-sensing properties was carried out using a two-probe technique by applying a constant 0.2 V bias to the films and measuring the through current with a picoammeter. Gas-sensing measurements were carried out by the flow-through method, working at a constant flow of 300 sccm in a thermostatic sealed chamber at room pressure under constant-humidity conditions (RH = 30% @ 20 °C). Controlled gas mixtures were obtained by using mass-flow controllers to mix flows from certified bottles. Sensor response was calculated as (*R*_gas_ – *R*_air_)/*R*_air_, denoted as Δ*R*/*R*, and (*G*_gas_ – *G*_air_)/*G*_air_, denoted as Δ*G*/*G* for oxidizing (N_2_O, NO_2_) and reducing gases (NH_3_), respectively. *R* and *G* represent the measured electrical resistance and conductance of the sample. Sensor response was measured at 190 °C operating temperature. We established through the TG measurements of functionalised ZnO samples, that the degradation of the organic capping layer of the nanowires and nanoparticles at this operating temperature was minimal.
